# Knowledge for games, games for knowledge: designing a digital roll-and-move board game for a law of torts class

**DOI:** 10.1186/s41039-016-0045-1

**Published:** 2017-01-26

**Authors:** Kok Yew Gary Chan, Swee Liang Tan, Khe Foon Timothy Hew, Bernie Grayson Koh, Li Siong Lim, Jose C. Yong

**Affiliations:** Singapore Management University, Singapore, Singapore

**Keywords:** Deep Learning, Constructivist Learning, Board Game, Educational Game, Qualitative Case Study

## Abstract

Game-based constructivist learning has gained considerable attention as educational institutions aim to move from traditional instructional teaching to interactive and collaborative methods. The question is less asked of *whether* games should be used in teaching but rather *how* games should be used to create deep learning in students. In light of this movement, the *Grade Inflation Game*, or *GIGAME*, was developed. This case study tackles the research question of how games may be designed to harness the benefits of constructivist learning. It first documents the conceptualization of GIGAME and next proposes theoretical frameworks that can be used to guide the development and evaluation of constructivist learning games. Through trial runs on two undergraduate law of torts classes, feedback was gathered from students and the instructor of these classes regarding their perceptions of GIGAME, including learning effectiveness and satisfaction. The feedback suggests that GIGAME can serve as a form of game-based constructivist learning as most students indicated that the game helped consolidate their course knowledge while having fun. These qualitative findings are useful for guiding the development of other games that similarly aim to promote socially interactive and constructivist learning environments. This case study recommends the use of GIGAME in classes, although refinements can still be made to its gameplay.

## Background

Many contemporary educational scholars have advocated for constructivist-driven teaching and learning, which has been shown to foster critical thinking and deeper learning (e.g., Kong and Song 2013; Lipman 1991; Otting and Zwaal 2007). At the same time, a wide body of research has shown that game-based learning is a highly effective method for various educational purposes (e.g., Shaffer 2006; Shih et al. 2013; Squire 2003). Researchers are increasingly identifying ways to employ the intrinsic educational traits of games to exploit constructivist learning environments (Jong et al. 2010). The question is less asked of *whether* games should be introduced into the classroom but rather *how* games should be developed and implemented to best harness deep learning in students.

In line with the progressive acceptance and use of games in contemporary classrooms, an electronic roll-and-move board game, named the *Grade Inflation Game* (*GIGAME*), was developed by Singapore Management University’s (SMU) Centre for Teaching Excellence (CTE) for a law of torts class based on principles that underlie constructivist learning, such as goals, social interaction, and autonomy. Given the growing interest in game-based learning, this case study aims to contribute to the knowledge on constructivist education by first describing the significance of constructivist and game-based learning, followed by outlining how GIGAME was designed with these constructivist principles in mind as well as the evaluation of its efficacy in classroom use.

This case study thus aims to answer the research question of how such games may be designed, implemented, and evaluated. Although the results of this case study are largely qualitative and exploratory, the insights gleaned are useful for guiding the development of other games that similarly aim to promote socially interactive and constructivist learning environments for students, as well as future research that aims to evaluate such educational games. The findings thus serve to encourage further research and work in this area, as well as to recommend that GIGAME and other similar games may be developed with the guidance of such theoretical frameworks and be utilized in classrooms by relevant educational practitioners to encourage deeper collaboration, inquiry, and learning in students.

### Constructivist learning

There are two overarching contrasting methods of educational practice. These are distinguished as the traditional standard pedagogical practice where the instructor plays an authoritative role in the instructional process and transmits his or her knowledge to learners in a uniform direction, against the critical reflective pedagogical practice where learners are encouraged to be participatory and reflective in an instructor-guided community of inquiry (Lipman 1991). Many modern educators and researchers advocate for a shift from the traditional standard practice, which is still dominantly practiced in most schools today, to the critical reflective practice (e.g., Kong 2015; Kong and Song 2013).

The theoretical underpinnings of a critical and reflective pedagogy are situated in social constructivist learning theories, which state that knowledge is best acquired when it is socially situated and constructed through an interaction of one’s own thoughts and experiences with others’ ideas (Otting and Zwaal 2007; Piaget 1964). Interactivity has been viewed as playing an essential role in the learning process as learners can engage with peers and instructors to exchange and share their knowledge (Bernard et al. 2009; Kong 2005), motivating learners to construct new knowledge and reorganize prior knowledge from this interaction process (Kang and Im 2013). A reflective environment that encourages autonomous interaction and collaboration between instructors and learners can improve learners’ knowledge acquisition and cognitive development greatly (Song and McNary 2011).

Following this social interactionist and constructivist learning philosophy as the basis of reflective and critical pedagogy, learners are encouraged to engage in active learning, including to discuss, argue, negotiate ideas, and collaboratively solve problems (Jonassen et al. 2003). The main role of instructors therefore shifts from being one that is instructional to one that deemphasizes hierarchy, designs and provides the learning context, and facilitates learning activities, allowing learners to be self-directed (Palincsar 1998). Studies have found that learners in a more interactive system learn better than and can also outperform those in a non-interactive system (Evans and Sabry 2003), and various technologies, such as simulations and games, have been increasingly recognized as effective means to create such socially interactive and constructivist learning environments (e.g., Okuda et al. 2009; Shang and Jong 2009; Shih et al. 2010).

### Game-based learning

For many disciplines, scholars, and practitioners, game-based learning is neither a new concept nor practice. Computer games are an important feature in the lives of many people today (Kirriemuir and McFarlane 2004), and the increasing ubiquity of digital technologies in modern society has significantly increased the ease of introducing games into classrooms (Squire 2003; Jong et al. 2010). Game-based learning can be done through either (1) education in games, which is the adoption of existing recreational games in the commercial market for educational use (e.g., Gee 2003), or (2) games in education, which is the development of educational games with constructivist learning paradigms in mind (e.g., Shaffer 2006).

Games are gaining wide recognition as an effective way to create socially interactive and constructivist learning environments, and Jong et al. (2010) suggest that there are at least three features of games that make them intrinsically educational. First, the motivational perspective argues that games are fun and engaging, which can sustain students’ motivation to learn, immerse them in goal-directed challenges, and foster deep learning (DeLisi and Wolford 2002; Bowman 1982; Malone 1981). Second, from a cognitive perspective, students engaged in complex gaming must proactively analyze the perceived information and context in the games, making students acquire new cognitive knowledge and skills and apply them to strategize and make decisions (Gee 2003). Lastly, the sociocultural perspective states that games that enable students to participate in communities help develop their social identity and also encourages sharing, discussing, and applying knowledge that has been co-constructed by peers (Cole 1996; Prensky 2006).

Along with this growth in the scholarship on game-based constructivist learning, a growing body of studies has sought to examine the effectiveness of games on educational and psychological outcomes across a wide variety of academic populations and subject matter (Wilson et al. 2009). For example, Papastergiou (2009) experimentally tested the learning effectiveness and motivational appeal of a computer game for learning computer science concepts on students from a Greek high school against a control group that did not use any gaming elements, and her results showed that the gaming approach was both more effective in promoting students’ knowledge of computer memory concepts as well as more motivational than the non-gaming approach. Ebner and Holzinger (2007) used a pre-test/post-test experimental design to assess the effectiveness of online games for a Master’s level civil engineering course and found that learning goals were met and students additionally reported increased enjoyment and engagement. In a comprehensive study, Blunt (2007) assessed three different games in three different education domains, namely Industry Giant II in business, Zapitalism in economics, and Virtual U in management. In each of these education domains, students provided with a game to facilitate learning scored significantly higher on tests (although these findings were only true for students below the age of 40, indicating that individual differences in receptivity to game-based learning and technology has a moderating influence). Game-based learning therefore allows students to explore social and cognitive learning contexts within games in an immersive, interactive, engaging, and sometimes realistic manner, thereby facilitating learning that is deeper and often more effective than traditional instructive teaching methods (Jong et al. 2010).

### The current study

The current study seeks to contribute both academic and practical insights to the scholarship and practice on education. Given the importance of harnessing the benefits of constructivist learning from games, the development and use of GIGAME in SMU’s classes serves as a useful source of information in two ways. First, GIGAME’s conceptualization and design is based on theoretical knowledge aimed at engaging students in an immersive gaming context to motivate socially interactive and constructivist learning. Such a theory-driven approach to GIGAME’s development is thus recommended for the development of other educational games, and the theoretical principles used in GIGAME’s development are fundamental and therefore also generalizable to the design of many other games for similar educational purposes.

Next, the efficacy of GIGAME was evaluated in a qualitative manner through two methods. First, students and faculty’s impressions of the game were collected in a general survey. Multiple evaluators who are well-versed with the research and trained with GIGAME were employed to analyze the responses and form a preliminary and general sense of how users perceive the game. Second, a highly useful framework developed to assess the educational efficacy of games by Gunter et al. (2008), called the Relevance, Embedding, Transfer, Adaptation, Immersion and Naturalisation (RETAIN) framework, was utilized to examine GIGAME’s effectiveness in facilitating constructivist learning. The same evaluators independently conducted assessments of GIGAME’s effectiveness across the various domains of RETAIN, and convergence in evaluators’ perceptions of GIGAME was considered as reasoned and acceptable ratings of the game. While it is recognized that the qualitative nature of these analytical methods and findings of this case study are indicative and not conclusive, qualitative case study methods still hold value in guiding our understanding of complex phenomena (Baxter and Jack 2008), especially when applied to constructivist research paradigms (Stake 1995; Yin 2003).

Therefore, the current study aims to meet the research objectives of how games may be developed, implemented, and evaluated for constructivist learning by first describing the sources of inspiration that led to the creation of GIGAME and discussing the theoretical principles that guide its design. Next, the findings from qualitative analyses of GIGAME’s efficacy in promoting constructivist learning will be described, which will indicate the extent to which the attempts to design and use GIGAME to achieve these educational objectives is effective.

## Case description

### GIGAME

CTE is a department within SMU that is tasked with enhancing the quality of teaching through sharing knowledge and building best practices. Amidst the growing research literature emphasizing the importance of constructivist learning and advocating the use of games in classrooms to create such learning environments, CTE sought to introduce games into SMU’s courses. GIGAME was conceptualized and developed by CTE after the use of games was deemed effective during some trial classes. This was part of CTE’s broader movement to encourage educational innovation in SMU through various forms of technology-enabled learning. This section describes how GIGAME was conceptualized, briefly details the simple gameplay of GIGAME, and highlights the key features of GIGAME that were designed to facilitate enhanced student learning based on psychological, motivational, and constructivist principles.

#### Background

In one SMU course, LAW104: Legal System, Legal Methods, and Analysis, a “Snakes and Ladders” style board game was trialled in 2011 and 2012. Aptly named *Snakes and Lawyers*, it was played in class by students to revise relevant concepts. The board game was beamed onto a stationary screen through an overhead projector, and the multiple choice questions relating to the topic on “The Singapore legal profession” were presented on PowerPoint slides one at a time. Students formed groups of four or five to play, and if a particular group answered the question correctly, it had another turn to roll a virtual dice and advance on the *Snakes and Lawyers* board. The group of students that managed to reach the top of the board first was declared the winner.

Despite the relatively unstructured and informal manner in which the game was played, the activity was generally well-received by the students. Students said that the game enabled them to interact with their peers, the competitive aspect of the game was engaging, and the questions were challenging. *Snakes and Lawyers* therefore hinted at the ability of such a game to foster a constructivist learning environment. However, some shortcomings of *Snakes and Lawyers* included the following:

1. Students were not able to play the game on their own outside of the classroom.
2. The questions and answers during the gameplay could not be tracked so students and the instructor had no effective way to recall what they had learned.
3. Some students expressed a lack of “game features” to further motivate them to play.
4. Each group knew its own position as represented by its piece on the board game but not the state of play among the competing groups.

CTE recognized the long-term potential of such a game: If properly designed, this game can be used easily, reliably, and sustainably in similar courses to engage students, motivate interactions with peers, and increase deeper learning. CTE was then tasked to develop an improved game that can be used for future courses.

#### Conceptualization and brief gameplay of GIGAME

The ideation process of the new game started with examining *Snakes and Lawyers* and its attendant shortcomings. An initial analysis of *Snakes and Lawyers* deemed the following as essential for the improved game:

1. Students should be able to consolidate their objective skills and knowledge while having fun.
2. Students should be able to play the game either on their own or interactively with their peers.
3. Students should be able to play the game either as an in-class or out-of-class activity.
4. Students should be able to reflect on their performance through a feedback system and competition with peers.

A thorough review of online games by CTE identified a similar game known as *Talisman* (https://boardgamegeek.com/boardgame/27627/talisman-revised-4th-edition), which has features such as distinct avatars, random elements, and a leaderboard, as an appropriate board game to model after for the new and improved game. In *Talisman*, players begin at the outer regions and try to progress towards the center of the board to reach the Crown of Command. Each player begins the game by selecting a character or avatar, and each avatar possesses different special abilities. One of the main goals of the game is to build up an avatar through conquests or destroying monsters so that it becomes strong enough to venture inward and eventually reach the Crown of Command. *Talisman*’s gameplay was simple yet immersive, which is ideal for game-based learning if only lacking the educational component.

**Fig. 1:**
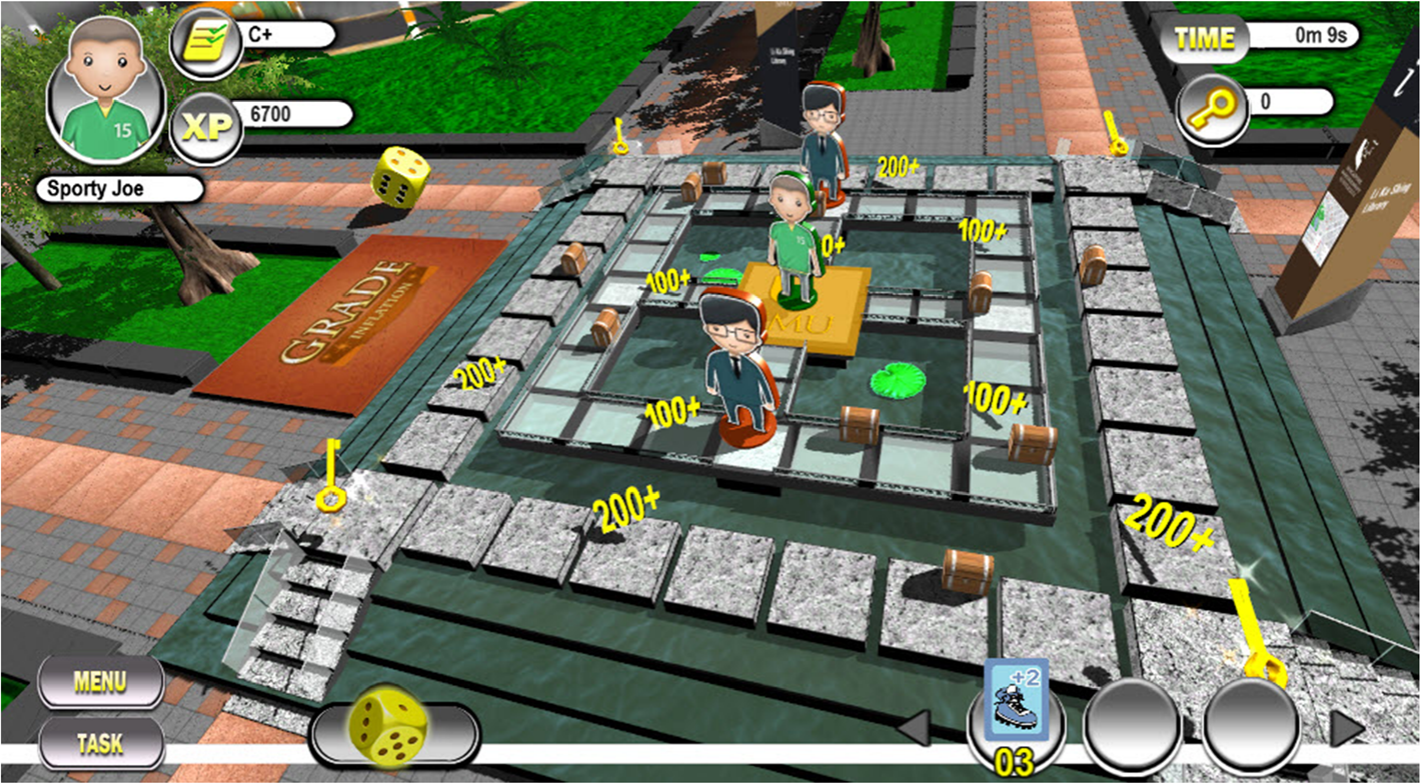
GIGAME was designed as an electronic roll-and-move style board game

**Fig. 2:**
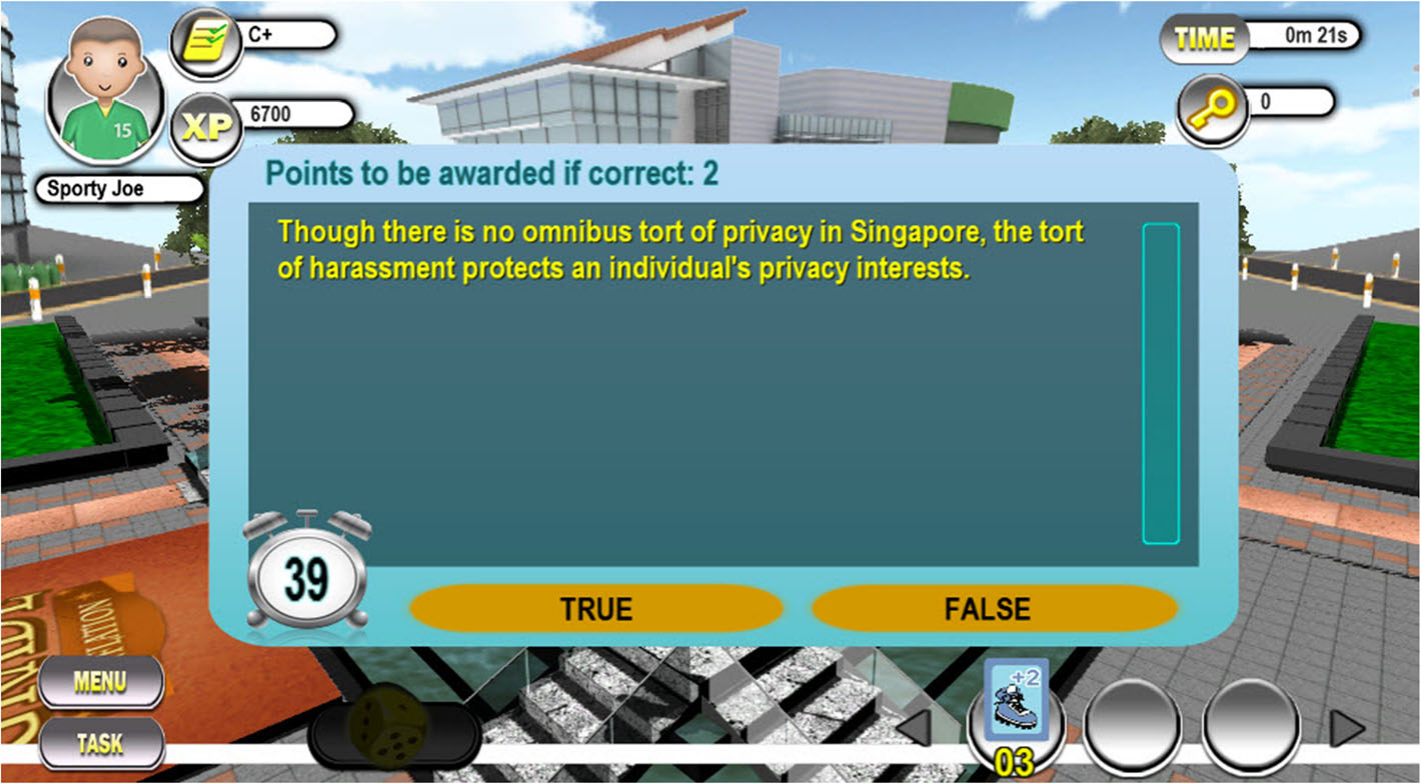
An example of a true/false question presented in GIGAME
  Each correct answer yields points which will inflate the player’s grade. Upon reaching a B grade, bridges will open allowing access to the outer area of the board. Players will then encounter more challenging questions to obtain A grades, which gives them access to the four keys needed to unlock the chest and win. Players can also obtain special cards with enhancements, such as to increase their movements and eliminate wrong answers. “Professors” roam the board challenging players with even more advanced questions. A leaderboard indicates each player’s rank compared to other players in real time.

Based on *Talisman*, the *Grade Inflation Game*, abbreviated as GIGAME, was conceptualized in January 2013 and its development was completed in March 2014. As shown in Fig. 1, the GIGAME was designed as an electronic roll-and-move style board game. Players in GIGAME first select a unique avatar and start the game in a “safe zone” on the board where there is a locked chest. Players begin with an equivalent of a C+ grade which is generally considered as a basic pass grade from which students should be encouraged to improve upon. In order to collect the keys to unlock the chest, players have to answer a series of questions in the form of true/false and multiple choice questions. As the game is aimed for use in the law of torts course, questions were related to law. CTE worked closely with the instructor, who has taught the module law of torts since January 2009, to rigorously develop a specific set of questions that could challenge the participants sufficiently. However, future conceptualizations of the game can be made to have questions relevant to other courses. All the questions require the application of concepts and/or principles covered in the course. We offer three examples, (A), (B), and (C) below.

(A) In which one of the following cases is there publication of defamatory statements?
  1. Statement that the plaintiff is highly corrupt found in an Internet website that is freely accessible to the public.
  2. Statement that the plaintiff is a liar in an email read by the plaintiff’s wife.
  3. Statement that the plaintiff is dishonest contained in a hyperlink.
  4. Statement that the plaintiff is a rapist found in an Internet website that can be searched by typing the plaintiff’s name.
  This question tests students’ understanding of the legal concept of “publication” in the tort of defamation, which is different from a layperson’s notion of a publication of a statement.
(B) Danny knew that Peter, a competitor in the same seafood business, was dependent on the same supplier as he was to provide abalone for the restaurant business. Danny informed the supplier, Sammy, that he would increase his demand for abalone by 50% as well as purchase large amounts of shark’s fin from Sammy every month, provided that Sammy will stop supplying abalone to Peter immediately. Sammy replied that under the existing contract with Peter, Sammy is entitled to terminate the contract with only three months’ notice. Danny said, “That’s too long. Stop supplying abalone to him as soon as possible and I will absorb your costs.” Sammy immediately stopped supplying abalone to Peter without giving any notice whatsoever. Peter had to quickly call up an alternative supplier of abalones who charged at least 20% more than Sammy. Which one of the following statements is correct?
  1. Peter is entitled to claim against Sammy for breach of contract and may claim from Sammy the additional costs of the abalones from the alternative supplier.
  2. Peter is not entitled to claim against Danny in tort concurrent with an action in breach of contract against Sammy.
  3. Peter is not entitled to claim against Danny for inducing breach of contract if Danny was not able to follow up on his promise to absorb Sammy’s costs of having to compensate Peter for breaching the contract prematurely.
  4. Danny is liable to Peter in deceit.
  The second question is based on a scenario which reveals a potential legal claim in tort law for inducing breach of contract. Students are expected to apply their knowledge of the legal requirements of the tort and the possible remedies to the facts described in the scenario.
(C) Though there is no omnibus tort of privacy in Singapore, the tort of harassment protects an individual’s privacy interests.
  1. True
  2. False
  Question example (C) assesses students’ knowledge of whether some torts may practically overlap. Figure 2 provides an example of how these questions are presented in GIGAME.

#### Theoretical underpinnings of GIGAME’s gameplay and features

GIGAME was developed with constructivist learning principles in mind. In reviewing the literature, various game-based learning researchers have emphasized a range of important psychological phenomena related to constructivist learning principles. As a constructivist-driven education involves learners actively engaging in a self-directed process of meaning and knowledge construction and inquiry (Piaget 1964), elements within constructivist learning tend to involve social interaction, motivation, and autonomy among learners. From this literature review, six relevant psychological domains were identified and consulted, namely goal-setting, self-efficacy, social comparison, self-determination, reinforcement, and emotion, in the use of points, the leaderboard, power cards, and other game features in GIGAME.

##### Goal setting

According to goal-setting theory, students’ motivation and learning can be promoted when their aspirations or goals are specific (Locke and Latham 1990). A goal is an outcome that an individual aims to achieve, and the word “game” itself implies that there is an “object of the game” (Malone 1981, p. 356). GIGAME is designed with various levels of goals to achieve. At a basic level, all players begin with a C+ grade at the start of the game, and subsequent correct answers to questions result in a player gaining more points, which would in turn improve his or her grade. At a broader level, players strive for higher grades in order to unlock their chests. Having points for grades gives participants a specific goal or target to aim for. However, a goal that is too simple or easily achieved will not be challenging to players. To create an intrinsically motivating experience, a goal should thus be moderately difficult or complex and stretch a player’s existing ability (Malone 1981). When this happens, a player is said to enter into a state termed by Csikszentmihalyi (1990) as flow or an intense and focused concentration on one’s activity in the present moment. CTE worked closely with the instructor to rigorously develop a specific set of questions that could challenge the participants sufficiently, and these questions required players to contemplate and apply the concepts learned in class. Further, players have to strategize in order to choose the best means to win at the game, such as opening their chests as quickly as possible or answering as many questions correctly as possible. These goals of varying complexity thus enhance players’ motivation and learning.

##### Self-efficacy

Self-efficacy is one’s belief in how well he or she can deal with prospective situations, thus determining the efforts and persistence that an individual would exert when facing obstacles (Bandura 1982). According to Richter et al. (2015), personal achievement is the most influential way to foster self-efficacy. The points’ system was specifically used as central to GIGAME’s gameplay with the aim of stimulating players’ self-efficacy through personal achievement by measuring an individual’s progression and providing direct feedback on performance (Gnauk et al. 2012).

##### Social comparison

According to social comparison theory, individuals evaluate their opinions and abilities by comparing with those of others (Festinger 1954). Two types of social comparison exist, namely upward identification, which refers to individuals comparing themselves with more competent people and believing that they can be as good as those better performers, and downward identification, which refers to individuals comparing themselves with others who are worse off so as to make themselves feel better (Suls et al. 2002). The leaderboard, which displays the rank an individual has achieved, can drive upward-identification comparison, which in turn can positively influence students to be more engaged in learning (Chen and Chen 2015). Moreover, in a game context, comparisons with other players based on “quantitative measurements” (e.g., the points’ system and the leaderboard) provoke competition, and such competition can serve as a challenge and motivation to master given tasks (Chang et al. 2013; Richter et al. 2015).

##### Self-determination

Self-determination theory states that people possess several psychological needs, and one of those is the need for autonomy (Deci and Ryan 2000). Autonomy refers to an individual’s need for freedom over one’s action. Game-based activities that offer individuals the freedom to choose their preferred strategies to complete the activity can address this need. Having a sense of autonomy can increase participants’ behavioral and emotional engagement (Skinner et al. 2008). In GIGAME, players are given the opportunity to make choices, such as whether they should answer the questions quickly and score bonus points, initiate a challenge against competitors, or use their power cards. For example, players can choose to either finish the game as quickly as possible and score the highest number of bonus points but attain less overall points from answering questions from the board or attempt to answer as many questions from the board as possible but end up with less bonus points based on time. The availability of choices serves to cater to an individual’s need for autonomy.

##### Reinforcement

Behavior can be motivated or forestalled by its resulting consequences, and a reinforcer is one of the many consequences that strengthen a behavior (Ferster and Skinner 1957). One of the most frequently used reinforcers is positive reinforcement. Positive reinforcement occurs when a new stimulus is presented as a consequence of a behavior, which consequently strengthens the behavior (Ferster and Skinner 1957; Woolfolk 1998). More specifically, game-based activities that give a reward (e.g., points) for every correct response represent one example of a consistent and continuous reinforcement. Continuous reinforcement was employed in GIGAME to increase students’ engagement in the gameplay as well as interest in using GIGAME, especially since this was the first time the game was introduced to the students. GIGAME also deducts points for wrong answers, which provides immediate feedback to players which can also contribute to their self-efficacy. In this way, players quickly know if they need to relearn the course content to better answer the questions.

##### Emotion

Fun activities naturally elicit emotional states, which in turn lead to increased attention and engagement (Hromek and Roffey 2009). One important focus in the design of the game is to make learning fun. Specific features are included in GIGAME to raise different types of emotions to motivate students to play. For example, the prospect of meeting the roaming Professors or the difficulties in getting the four keys can rouse various emotions, such as excitement and frustration. The multiplayer classroom mode can evoke emotions that come with collaboration or competition within and between teams. According to Lazzaro (2012), there are four types of fun, namely Easy Fun, Hard Fun, Serious Fun, and People Fun. These four types of fun generate a wide range of emotions and enhance the game experience. For instance, Easy Fun emotions maintain player attention without challenge through novelty and inspiring fantasy. Hard Fun creates challenges with strategies and puzzles. Serious Fun teaches or accomplishes real work. People Fun motivates group interaction and interpersonal relations and creates social emotions between players. These emotions have a significant effect on enjoyment, attention, memory, learning, and performance.

Taken together, the development of GIGAME is guided by these underlying principles of constructivist learning. Student interest in and motivation to play the game is fostered by having specific and challenging goals through the points’ system, which also serves as a positive reinforcement and enables students to experience progression through the game. The leaderboard fosters a competitive atmosphere where students are engaged in the objective of outperforming others. Students are also engaged when given the opportunity to strategize and make decisions, as well as when they experience heightened emotional states during gameplay. When playing GIGAME, these gameplay features are aimed at increasing students’ immersion in the game, thereby increasing their attention to and memory of important course concepts.

### Method

While the development of GIGAME is driven by sound theoretical principles, it is not immediately certain that theory translates into practice. In order to ascertain the efficacy of GIGAME as a game that purports to facilitate constructivist learning, two trials of GIGAME and qualitative analyses were conducted. The authors of the current study tested GIGAME on students from two law of torts classes as well as trained evaluators. The trained evaluators provided a reasoned qualitative assessment of GIGAME’s effectiveness based on feedback from users (students and the instructor) as well as the RETAIN framework for assessing educational efficacy.

Gunter et al.’s (2008) RETAIN framework consists of six components that critically address the different elements in learning and the degree to which a game helps students to construct knowledge. These components are relevance, embedding, transfer, adaptation, immersion, and naturalization. Relevance refers to whether materials in the activity are presented in a coherent way that is related to the course material and students’ learning style. Embedding refers to how well the academic content fits into the gameplay or storyline. Transfer refers to how well players can use knowledge from various areas and apply them. Adaptation refers to how well a game enables students to create new knowledge to make sense of something within the activity that does not fit their current ideas or understanding. Immersion refers to how intellectually engaged or invested the player is within the game or activity. Lastly, naturalization refers to how well the game encourages players to develop habitual and spontaneous use of information derived within the game or activity. This framework was specifically selected for analyzing GIGAME as it is widely regarded in the research literature as the main evaluation criteria of educational efficacy. Its construct is also anchored on several seminal theories that are highly relevant to interaction, motivation, engagement, and constructivism, such as Keller’s (1987) ARCS (attention, relevance, confidence, and satisfaction) model of motivation and Gagné et al.’s (1992) nine events of instructions (gain attention, inform of objectives, stimulate recall, present stimulus/lesson, provide learner guidance, elicit performance, provide feedback, assess performance, and accommodate retention and transfer). The RETAIN framework is also a simple and grounded model which evaluators can use with ease.

Qualitative case study methods typically produce findings that are preliminary and indicative. However, rigorous qualitative case studies have also afforded researchers rich opportunities to explore or describe highly contextualized phenomena using a variety of data sources. Qualitative research allows researchers to explore individuals or organizations (both simple and complex) dynamically and is especially apt for examining research questions with constructivist underpinnings since qualitative case studies support the deconstruction and the subsequent reconstruction of various phenomena (Stake 1995; Yin 2003). According to Yin (2003), a qualitative case study design should be considered when (a) the focus of the study is to answer “how” and “why” questions, (b) the behavior of those involved in the study cannot be manipulated, (c) contextual conditions that are relevant to the phenomenon under study should be explored, or (d) the boundaries between the phenomenon and context are unclear. The exploratory nature of this study of GIGAME, such as the effectiveness of eliciting constructivist learning from its theoretically guided development, is especially pertinent to (a) and (b), and a qualitative assessment is thus justified. That said, these findings can be most accurately described as users’ and evaluators’ *perceived* effectiveness of GIGAME, and more studies should be conducted to further refine the insights gained here.

#### Procedure

In the last week of the semester in March 2014, GIGAME was introduced to 76 students in two law of torts classes. The game was played as a form of revision of materials. Each class was divided into eight groups with each group represented by an avatar. Each group took turns to roll the dice, move their avatars, and answer questions. The trial of GIGAME in each class ended after approximately 60 min which was the time allotted for the lesson. After the game ended, an online survey was sent to all participating students, and a total of 64 students responded to the survey yielding a participation rate of 84.2%.

#### Evaluators

Three evaluators were employed to examine the efficacy of GIGAME through the user feedback and the RETAIN framework assessment methods, which will be described further next. These evaluators are research staff of CTE who are experienced with research and analysis methods as well as the academic literature on constructivist and game-based learning and are also trained with playing GIGAME. The evaluators provided their initial assessments independently so as to ensure that unbiased judgments were first made. There was overall independent agreement on most of the assessments. Any disagreements, though minor, were rigorously discussed by all evaluators so as to reach a reasoned and valid consensus.

#### Assessment measures

##### User feedback

Both students and the instructor of the law of torts course were asked to provide feedback and comments about GIGAME. Students were given an online survey to complete after playing the game. They were ensured that their responses were confidential and they would remain anonymous, so they could answer honestly. The survey consisted of a total of 7 face-valid questions adapted from the assessment instruments used by other researchers to similarly measure student feedback and learning outcomes (e.g., Papastergiou 2009). Due to classroom and logistical constraints, the survey was designed to be short and students only could respond whether they agreed (e.g., “agree,” “yes”), disagreed (e.g., “disagree,” “no”), or neither agreed nor disagreed (e.g., “neither agree nor disagree,” “neutral”) with the questions. Of these questions, six focused on a particular aspect of GIGAME, such as the perceived learning effectiveness, gameplay technical issues, and satisfaction, followed by one which asked for general comments to capture students’ broad perceptions about the game and to provide their reasons for those perceptions. For each of the GIGAME-specific questions, students were also solicited for an elaboration of their answer. For example, when asked about whether they thought GIGAME was effective as a learning tool, students were asked, “Was *GIGAME* effective in helping you learn?” followed by “Please state your reasons.” Students could therefore further elaborate on their perceptions if they had more to say about a particular issue, although it is not mandatory to do so. Evaluators then determined the extent that students felt either positively or negatively about a particular issue. The instructor also was asked for his open-ended comments about GIGAME. These general feedback and comments are aimed at capturing preliminary impressions of GIGAME, and the evaluators formed a reasoned assessment of GIGAME from the user’s perspective. In addition, these responses also serve as useful information that helped guide the evaluators’ assessment of GIGAME using the RETAIN framework.

##### RETAIN framework

Evaluators used the RETAIN framework (Gunter et al. 2008) to determine the extent to which the game helped students engage in the acquisition and construction of knowledge. These components are relevance, embedding, transfer, adaptation, immersion, and naturalization and will be explicitly stated in the analysis section. However, the evaluators did not use the weighting factor suggested by Gunter et al. (2008) for each of the components in the RETAIN framework, as GIGAME was designed for a different purpose and hence the components are meaningful in their own way. Instead, a likert-type rating metric suggested by Ulicsak and Wright (2010) is a viable alternative and was used to rate the effectiveness of GIGAME in terms of delivering the RETAIN objectives. The ratings range from 1, which approximately translates to very little, to 4, which translates to very much. As each component focuses on a distinct aspect of constructivist learning, what the ratings meant were carefully adjusted to improve their validity after several rounds of discussion between evaluators to arrive at a consensus. Evaluators also used the user feedback to guide their rating decisions under the RETAIN framework. We estimated the convergence between evaluators by estimating the mean *r*_wg_ coefficient and coefficient alpha index, as well as two intraclass correlations. All four measures were acceptable, suggesting adequate levels of agreement and reliability (*r*_wg_ = .85; *α* = .83; *ICC*_*1*_ = .67; *ICC*_*2*_ = .80).

## Discussion and evaluation

### Analysis and results

This section details the analyses conducted and the findings gathered from the two forms of assessments. First, the evaluators present a broad impression of GIGAME’s effectiveness from the point of view of users. This is followed by a more rigorous qualitative evaluation of GIGAME’s educational efficacy using the RETAIN framework.

#### User feedback (i): students

After playing the game, students were administered the online survey. The survey included seven questions that captured general perceptions of GIGAME as well as any further details on why they feel the way they do, enabling the authors to form a preliminary impression of the game.

##### Gameplay design

Sixty-three students (98.4%) agreed that the design of the game was appealing and motivating. Most students provided elaborative remarks such as “I liked the smooth interface of the game” and “Very engaging and appealing; the interface was designed very well.” Comments emphasized that the graphics were good and that the gameplay was not too complicated.

##### Perceived learning effectiveness

Fifty-nine students (92.2%) rated the game to be effective in helping them to learn. Some comments include, “The questions incorporated into the game were challenging, which made the game useful as a learning tool.” Students generally indicated that they will be able to remember the course concepts better since the experience of playing the game would aid in their memory retention. For students who felt that GIGAME was not very useful as a learning tool, they said that “class discussions are more effective.”

##### Perceived change in knowledge after the activity

Thirty-seven students (57.8%) reported a perceived increase in knowledge after the activity. Some students stated that the discussions among peers made them rethink concepts that they had misunderstood. For instance, one student said, “The game helped to further my understanding of the finer points of tort law and was very helpful in clarifying certain misconceptions.”

##### Technical problems encountered

Forty-three students (67.2%) reported technical problems. Most of the students who stated that they encountered technical difficulties pointed out that the text font was hard to read. Other problems encountered also relate to blocked views and the game either lagging or hanging. One student reported experiencing dizziness.

##### Satisfaction from the game

Sixty-four students (100%) agreed that they were satisfied with GIGAME as a learning activity.

##### Suggested improvements

Twenty-eight students (43.8%) requested for features that would enable them to review the questions, answers, and explanations so that they could learn from their mistakes. Seven students (10.9%) commented that some elements of the game were unnecessarily time-consuming (e.g., the time lag of dice rolling and movement of the avatars and the constant popping up of the instruction panel).

##### Other comments

Most students did not provide further comments. Nineteen students (29.7%) remarked that the activity was a fun way to revise. A small minority (three students) had opposing views on how and when they would play GIGAME, stating that either they will only play it in class and “will not play it at our own time” or “class time should be used for discussing cases” and should not be used for playing games.

#### User feedback (ii): instructor

The instructor was also solicited for his feedback on GIGAME after the classes ended. He said, “Students could not see the entire *Snake and Lawyers* game unfolding and the state of play among the competitors. They could only see their own position on the gameboard. For GIGAME, everyone can view the state of play contemporaneously which enhances the competitive element. Many students found GIGAME interactive and fun. It also allowed them to assess their own level of knowledge on the law of torts at the end of the course. When I revealed the multiple choice answers, there were at least a few eureka moments for certain groups of students.” The instructor was satisfied with the outcomes of the game, and he felt that it served his need for a fun and interactive activity that promoted knowledge retention and application.

#### Summary of the overall user perceptions of GIGAME

Based on the feedback of the students and the instructor, the evaluators concluded that GIGAME appears to be generally appealing as a form of game-based learning. Majority of the students said that the game helped consolidate their course skills and knowledge while having fun, and approximately 30% of the students mentioned that the activity was a fun way to revise. Students can also engage interactively with the game as well as with their peers as the game can be played both during and outside of class, and students can reflect on their own performance through feedback and competition with peers. Technical problems were mostly cited as display and slowness issues; these are not severe and can easily be improved in later development iterations of the game. Finally, every student indicated that they were satisfied with the game.

It is interesting to note that some students prefer to play the game together as a class, with comments such as “will most probably not play it at our own time” whereas some stated that the game could be used as an after class activity, as “class time should be used for discussing cases” and “class discussions are more effective.” Hence, it may be of interest for future research to identify the effects of individual preferences, such as how different personality types affect their receptivity towards game-based learning. Further research can also be done to determine how differently students respond to the game as individual players and as team players. In addition, future studies could be conducted on a larger scale to investigate the effectiveness of games as a revision activity, an activity for teaching of new concepts, or part blended learning where students could learn new concepts outside the classroom.

Overall, the feedback and comments from both students and the instructor showed that GIGAME had addressed the shortcomings of the original *Snake and Lawyers* game and created an atmosphere of fun learning in a university setting. The generally positive feedback from both students and instructor affirmed the effectiveness of the use of games in the classroom (Shang and Jong 2009).

##### GIGAME’s effectiveness based on the RETAIN framework

The evaluators carefully assessed GIGAME’s ability to create a constructivist learning environment such that students could autonomously interact, acquire knowledge, and construct new knowledge using the RETAIN framework. Table 1 details this analysis. The left column lists the six constructivist components of the framework, a description of each component, and the rating scale used to assess the extent to which GIGAME meets that particular component. The Likert scale ranges generally from 1 (the lowest) to 4 (the highest), although it will be specified what this scale means within the context of that particular component. The right column provides the evaluators’ ratings, an explanation for how that rating was chosen, and reliability scores.

**Table 1:** Assessment of GIGAME using the RETAIN framework

Components of the RETAIN framework	Assessment of GIGAME
Relevance ● Whether materials are presented in a way that is relevant to players, their needs, and their learning styles ● Whether the instructional units are relevant to one another, linked together, and built upon previous work as the player’s skill increases Rating scale 1—little stimulus for learning 2—limited educational focus, some irrelevant content 3—learning objectives are defined, and interest is created 4—game is highly relevant to learners, and challenges are adequate for learning	Evaluators’ rating: 4 The objective of revising various concepts in the law of torts is clear. The design of the game is attractive to the undergraduate audience; 63 out of 64 students agreed stated that the design of the game was appealing and highly motivating to use, and 62 out of 64 students agreed that the game and the whole activity were engaging. Questions posed were carefully designed and selected and are related to the concepts taught in the course, so that students can link them with their prior knowledge and build upon them to answer new application questions.
Embedding ● How closely the academic content is coupled with the gameplay, fantasy, or story content (i.e., the narrative structure, storylines, player experience, dramatic structure, fictive elements) Rating scale 1—learning content disrupts play 2—learning is exogenous to (or “outside”) the fantasy context 3—learning is somewhat linked to the storyline and includes intellectual challenges and problems 4—content is highly endogenous to the fantasy of the storyline and fully involves the learner	Evaluators’ rating: 2 In the initial design, the game was set in the context of achievements and grades in SMU. This presents a highly identifiable scenario for students. Given that this game is designed for a law of torts course, alternative designs can be further considered, such as a courtroom-like setting if the embeddedness of the game is to be enhanced.
Transfer ● Whether the player is urged to use previous knowledge and apply it to another area or level Rating scale 1—levels of challenge are not mapped to objectives 2—levels of challenge are similar, with some useful content 3—easy progress through levels via active problem solving, with some higher levels of knowledge being transferable 4—challenging and authentic situations that simulate reality and require knowledge application from various areas	Evaluators’ rating: 2 The game comprises largely of true/false and multiple choice questions, and the knowledge transfer applies primarily to objective, factual, and law-based knowledge. The structure of GIGAME’s gameplay limits the ability of players to transfer knowledge from other areas to the game or from the game to other areas.
Adaptation ● Whether players are compelled to change or create new knowledge to deal with or make sense of something that does not fit their existing ideas and understanding, often as a consequence of transfer Rating scale 1—information is unstructured and players cannot engage in interactive learning 2—builds upon existing cognitive structures, engages players in some cognitive conflict and reconstruction 3—players are encouraged to go beyond given information; old schemas are identified and adapted to new situations 4—learning becomes an active process that naturally integrates prior knowledge	Evaluators’ rating: 2 for single player mode; 3 for classroom mode The game may not directly promote students’ adaptation of knowledge because the questions, which serve as the challenges within the game, are in multiple choice or true/false question format. However, when the game is played in classroom mode, some of the questions are open ended, and hence, GIGAME garners a higher adaptation rating here. More authentic real-life problems that encourage the player to discover for themselves new concepts based on their prior knowledge may be incorporated in future improvements to GIGAME.
Immersion ● Whether the player is engaged and investing intellectually in the context of the game Rating scale 1—no formative feedback and little active participation 2—elements of play are not in sync with learning objectives and players are not engaged 3—learners are involved cognitively, physically, and emotionally 4—favors belief creation and includes opportunities for reciprocal action	Evaluators’ rating: 3 The game requires players to be fully engaged and conversant with targeted academic content and questions. Taking on avatar roles with special powers and landing on the same square as the “Professor” character can enhance players’ experience and motivate players to complete the game. To some extent, the game is designed to enable the players to be immersed in the game and achieve a state of flow (Csikszentmihalyi 1990). As players are observed to be involved in the game with a clear set of goals and progress, the game has clear and immediate feedback, there is balance between the perceived challenges of the task at hand and players’ own perceived skills, and players are likely to be in some state of flow and therefore immersed (Elliot and Dweck 2005). The high satisfaction ratings given by students also validates this view, as experiencing a state of flow in an activity also tends to increase satisfaction with that activity (Csikszentmihalyi 1990). However, a rating of 4 was not given as the evaluators deemed the game not complex enough for players to feel completely sucked into the game.
Naturalization ● How well players develop habitual and spontaneous use of information is derived within the game Rating scale 1—little opportunity for mastery of knowledge and skills 2—replay is encouraged to improve speed of processing 3—encourages synthesis of elements and judgment 4—players become efficient content users and spontaneously use acquired knowledge	Evaluators’ rating: 2 Repeated playing, if done purposefully, enables content to be ingrained in players’ habitual thinking. Formative feedback to remediate misconceptions of learning may be added to enhance the learning experience of GIGAME. If students feel that they are learning, they will be encouraged to play repeatedly. More variations to the gameplay (such as new questions, bonus challenges) can add curiosity and context variation to the game, encouraging students to revisit (and internalize) the academic content.

In summary, GIGAME scored highest in terms of relevance, which pertains to how well materials are presented so that they are relevant to the players’ learning needs, and how well linked the instructional units are with previous work as players’ skills increase. This is due to the careful development of questions designed to challenge players within the law of torts context. All of the other components within the RETAIN framework received satisfactory ratings, and there were no components that were rated 1, indicating that the theoretically guided development of GIGAME did translate into some extent of effectiveness in promoting constructivist learning. At the same time, these are not perfect and GIGAME can stand to benefit from refinements in future developments. Some components can achieve different ratings depending on the gameplay mode. In the case of GIGAME, its ability to induce adaptation was rated either 2 or 3 depending on whether the game was played in single player mode or classroom mode. On the whole, these limits may be owed to GIGAME’s simple game design, but such a design is sufficient for general classroom use.

GIGAME was conceptualized after trials with *Snakes and Lawyers* found that, despite its shortcomings, students were receptive to electronic roll-and-move board-style games. The development of GIGAME was driven by ideas that underlie constructivist learning. Two qualitative assessments were conducted to examine the efficacy of GIGAME in promoting constructivist learning and determine if the theory-driven development of GIGAME translated into real desired outcomes in practice. Overall, the analyses showed that GIGAME did overcome the shortcomings of *Snakes and Lawyers* and went further by garnering overall positive feedback from users. Importantly, students felt that the game was fun and engaging and facilitated their learning of course material. GIGAME successfully created an immersive environment which increased students’ satisfaction while playing the game. Gunter et al.’s (2008) RETAIN framework was used to guide the trained evaluators’ analysis of GIGAME more rigorously and help identify its strengths and the areas it can potentially improve. The findings reveal that the theoretical framework suggested by the current study, which recommends focusing on players’ goal setting, self-efficacy, social comparison, self-determination, reinforcement, and emotions, is useful for the development of games aimed at harnessing the benefits of constructivist learning.

#### Limitations and future directions

One potential limitation of this study is that it uses a qualitative research paradigm to analyze the effectiveness of GIGAME. While qualitative case study researchers have a strong case for how qualitative research paradigms still serve as rich sources of data (e.g., Baxter and Jack 2008), especially when applied to constructivist research questions (e.g., Yin 2003), critics of qualitative case study methods often cite subjectivity in the analyses, and the effectiveness that is assessed in the current study can only be regarded as perceived effectiveness, rather than real effectiveness. Further studies should be conducted to supplement the findings of this study. An important contribution of this case study is that it documents the process of game conceptualization and makes a reasoned and valid suggestion for how educational games may be developed and evaluated. Future research should involve controlled experimental designs, which include treatment and control groups, to rigorously determine the actual effectiveness of the use of GIGAME and other educational games. Important dependent variables, including memory retention, knowledge increments, and actual academic performance, can then be assessed, thereby strengthening this research.

The effectiveness of GIGAME should be examined with other student populations, including those from other disciplines as well as those from other universities, to determine if the effectiveness achieved in this study’s trials are replicable in and generalizable to other populations. Future research can also be conducted that benefits our broader understanding of games and psychology. For instance, student feedback for GIGAME revealed that some students preferred to play the game in class, whereas some felt that games should be kept out of the classroom. Future studies can also be conducted to investigate the effectiveness of games as a revision activity, an activity for teaching of new concepts, or part blended learning where students could learn new concepts outside the classroom.

Aside from minor technical issues raised by some students, such as the gameplay speed or font display, the RETAIN framework highlighted various other important conceptual areas that GIGAME could improve on. Further development iterations of the game should strive to improve the embeddedness of course content with the game, such as by exploring alternative designs including courtroom-like settings or other settings that are relevant to the course content. More authentic real-life problems that encourage players to discover for themselves new concepts based on their existing knowledge may be incorporated, so that players may engage more in adapting and transferring their knowledge across different but related domains. For example, questions that test the same concepts but in new situations can be designed and sequenced in a way to scaffold learning based on prior questions. Variations to the gameplay (such as new questions and bonus challenges) can add to players’ curiosity and experience of novelty within GIGAME. Baker et al. (2010) and Wang et al. 2013 also suggest that cognitive styles and pre-existing abilities can influence players’ tolerance of frustration and learning achievement in interactive games. These individual differences can be taken into account so that further developments of the game can fit the needs of players. Lastly, real-time formative feedback can be introduced into the gameplay so that students can learn more effectively. The nature of digital games allows these features to be changed and updated with relative ease, and this works to the advantage of game developers and course instructors.

## Conclusions

In summary, careful assessment and rating by trained evaluators showed that GIGAME fares decently for a new and simple game, which warrants its use in relevant courses as well as further development to refine its gameplay to create constructivist learning environments. This case study provides preliminary evidence supporting the view that game-based characteristics can enhance the constructivist learning experience of students. The theoretical frameworks utilized in this study are shown to be useful for guiding developers and instructors about characteristics in games that can engage and deepen learning, such as to make goals specific or to make the game immersive and aid in the transfer of learning. GIGAME is thus recommended for further use in classes, although refinements can still be made to its gameplay.

## References

[CR1] Bandura A (1982). Self-efficacy mechanism in human agency. American Psychologist.

[CR2] Baker RSJD, D’Mello SK, Rodrigo MMT, Graesser AC (2010). Better to be frustrated than bored: the incidence, persistence, and impact of learners’ cognitive affective states during interactions with three different computer-based learning environments. International Journal of Human-Computer Studies.

[CR3] Baxter P, Jack S (2008). Qualitative case study methodology: study design and implementation for novice researchers. The Qualitative Report.

[CR4] Bernard RM, Abrami PC, Borokhovski E, Wade CA, Tamim RM, Surkes MA (2009). A meta-analysis of three types of interaction treatments in distance education. Review of Educational Research.

[CR5] Blunt R (2007). Does game-based learning work? Results from three recent studies. Proceedings of the Interservice/Industry Training, Simulation, and Education Conference.

[CR6] Bowman RF (1982). A Pac-Man theory of motivation: tactical implications for classroom instruction. Educational Technology.

[CR7] Chang B, Chuang MT, Ho S (2013). Understanding students’ competition preference in multiple-mice supported classroom. Educational Technology and Society.

[CR8] Chen YH, Chen PJ (2015). MOOC study group: facilitation strategies, influential factors, and student perceived gains. Computers and Education.

[CR9] Cole M (1996). Cultural psychology: a once and future discipline.

[CR10] Csikszentmihalyi M (1990). Flow.

[CR11] Deci EL, Ryan RM (2000). The “what” and “why” of goal pursuits: human needs and the self-determination of behavior. Psychological Inquiry.

[CR12] DeLisi R, Wolford JL (2002). Improving children’s mental rotation accuracy with computer game playing. The Journal of Genetic Psychology.

[CR13] Ebner M, Holzinger A (2007). Successful implementation of user-centered game based learning in higher education: an example from civil engineering. Computers and Education.

[CR14] Elliot AJ, Dweck CS (2005). Handbook of competence and motivation.

[CR15] Evans C, Sabry K (2003). Evaluation of the interactivity of web-based learning systems: principles and process. Innovations in Education and Teaching International.

[CR16] Ferster CB, Skinner BF (1957). Schedules of Reinforcement.

[CR17] Festinger L (1954). A theory of social comparison processes. Human Relations.

[CR18] Gagné RM, Briggs LJ, Wager WW (1992). Principles of instructional design.

[CR19] Gee J (2003). What video games have to teach us about learning and literacy.

[CR20] Gnauk B, Dannecker L, Hahmann M (2012). Leveraging gamification in demand dispatch systems.

[CR21] Gunter GA, Kenny RF, Vick EH (2008). Taking educational games seriously: using the RETAIN model to design endogenous fantasy into standalone educational games. Educational Technology Research and Development.

[CR22] Hromek R, Roffey S (2009). Promoting social and emotional learning with games. Simulation and Gaming.

[CR23] Jonassen D, Howland J, Moore J, Marra R (2003). Learning to solve problems with technology: a constructivist perspective.

[CR24] Jong MS, Shang J, Lee F, Syed M (2010). Constructivist learning through computer gaming. Technologies shaping instruction and distance education: new studies and utilizations.

[CR25] Kang M, Im T (2013). Factors of learner-instructor interaction which predict perceived learning outcomes in online learning environment. Journal of Computer Assisted Learning.

[CR26] Keller JM (1987). Development and use of the ARCS model of instructional design. Journal of Instructional Development.

[CR27] Kirriemuir J, McFarlane A (2004). *Literature review in games and learning* (no. 8).

[CR28] Kong SC, Lee FL, Yeung H (2005). A research framework for developing interactive learning environment. Research handbook on computers in education: methodology and case study.

[CR29] Kong SC (2015). An experience of a three-year study on the development of critical thinking skills in flipped secondary classrooms with pedagogical and technological support. Computers and Education.

[CR30] Kong SC, Song Y (2013). A principle-based pedagogical design framework for developing constructivist learning in a seamless learning environment: a teacher development model for learning and teaching in digital classrooms in school education. British Journal of Educational Technology.

[CR31] Lazzaro N, Jacko JA (2012). Why we play: affect and the fun of games. Human-computer interaction handbook: fundamentals, evolving technologies, and emerging applications.

[CR32] Lipman M (1991). Thinking in education.

[CR33] Locke EA, Latham GP (1990). A theory of goal setting and task performance.

[CR34] Malone TW (1981). Toward a theory of intrinsically motivating instruction. Cognitive Science.

[CR35] Okuda Y, Bryson EO, DeMaria S, Jacobson L, Quinones J, Shen B, Levine AI (2009). The utility of simulation in medical education: what is the evidence?. Mount Sinai Journal of Medicine: A Journal of Translational and Personalized Medicine.

[CR36] Otting H, Zwaal W, McCuddy MK, Van-den-Bosch H, Martz WB, Alexei AV, Morseb KO (2007). The identification of constructivist pedagogy in different learning environments. The challenges of educating people to lead in a challenging world.

[CR37] Palincsar AS (1998). Social constructivist perspectives on teaching and learning. Annual Review of Psychology.

[CR38] Papastergiou M (2009). Digital game-based learning in high school computer science education: impact on educational effectiveness and student motivation. Computers and Education.

[CR39] Piaget J (1964). Development and learning. Journal of Research in Science Teaching.

[CR40] Prensky M (2006). Don’t bother me mom—I’m learning.

[CR41] Richter G, Raban DR, Rafaeli S, Reiners T, Wood LC (2015). Studying gamification: the effect of rewards and incentives on motivation. Gamification in education and business.

[CR42] Shaffer D (2006). How computer games help children to learn.

[CR43] Shang JJ, Jong MSY (2009). The educational value of games. Journal of Distance Education.

[CR44] Shih JL, Chuang CW, Hwang GJ (2010). An inquiry-based mobile learning approach to enhancing social science learning effectiveness. Educational Technology and Society.

[CR45] Shih JL, Hsu YJ, Wang YJ (2013). Rational emotive path: 3D game as the emotion analysis tool for counselling purposes. International Journal of Arts and Technology.

[CR46] Skinner E, Furrer C, Marchand G, Kindermann T (2008). Engagement and disaffection in the classroom: part of a larger motivational dynamic?. Journal of Educational Psychology.

[CR47] Song L, McNary SW (2011). Understanding students’ online interaction: analysis of discussion board postings. Journal of Interactive Online Learning.

[CR48] Squire KR (2003). Video games in education. International Journal of Intelligent Games and Simulation.

[CR49] Stake RE (1995). The art of case study research.

[CR50] Suls J, Martin R, Wheeler L (2002). Social comparison: why, with whom, and with what effect?. Current Directions in Psychological Science.

[CR51] Ulicsak, M, & Wright, M (2010). *Games in education: serious games*. A Futurelab literature review. Futurelab. United Kingdom, June. Retrieved from http://media.futurelab.org.uk/resources/documents/lit_reviews/Serious-Games_Review.pdf. Accessed 15 Aug 2016.

[CR52] Wang, MY, Tang, DL, Kao, CT, Sun, VC (2013). Banner evaluation predicted by eye tracking performance and the median thinking style, in Design, User Experience, and Usability: Health, Learning, Playing, Cultural, and Cross-Cultural User Experience, ed Marcus A., editor. Las Vegas: Springer Berlin Heidelberg, 129–138.

[CR53] Wilson KA, Bedwell WL, Lazzara EH, Salas E, Burke CS, Estock JL, Orvis KL, Conkey C (2009). Relationships between game attributes and learning outcomes: review and research proposals. Simulation and Gaming.

[CR54] Woolfolk A (1998). Educational psychology.

[CR55] Yin RK (2003). Case study research: design and methods.

